# m6A demethylase FTO promotes tumor progression via regulation of lipid metabolism in esophageal cancer

**DOI:** 10.1186/s13578-022-00798-3

**Published:** 2022-05-14

**Authors:** Xiaoran Duan, Li Yang, Liuya Wang, Qinghua Liu, Kai Zhang, Shasha Liu, Chaojun Liu, Qun Gao, Lifeng Li, Guohui Qin, Yi Zhang

**Affiliations:** 1 Biotherapy Center and Cancer Center, The First Affiliated Hospital of Zhengzhou University, Zhengzhou, 450052 Henan P.R. China; 2 Internet Medical and System Applications of National Engineering Laboratory, Zhengzhou, 450052 Henan P.R. China; 3 State Key Laboratory of Esophageal Cancer Prevention & Treatment, Zhengzhou, 450052 Henan P.R. China; 4 Henan Key Laboratory for Tumor Immunology and Biotherapy, Zhengzhou, 450052 Henan P.R. China

**Keywords:** Demethylase FTO, m6A, Lipid Metabolism, Esophageal cancer

## Abstract

**Background:**

Epitranscriptomics studies have contributed greatly to the development of research on human cancers. In recent years, N6-methyladenosine (m6A), an RNA modification on the N-6 position of adenosine, has been found to play a potential role in epigenetic regulation. Therefore, we aimed to evaluate the regulation of cancer progression properties by m6A.

**Results:**

We found that m6A demethylase fat mass and obesity-associated protein (FTO) was highly expressed in esophageal cancer (EC) stem-like cells, and that its level was also substantially increased in EC tissues, which was closely correlated with a poor prognosis in EC patients. FTO knockdown significantly inhibited the proliferation, invasion, stemness, and tumorigenicity of EC cells, whereas FTO overexpression promoted these characteristics. Furthermore, integrated transcriptome and meRIP-seq analyses revealed that *HSD17B11* may be a target gene regulated by FTO. Moreover, FTO promoted the formation of lipid droplets in EC cells by enhancing HSD17B11 expression. Furthermore, depleting YTHDF1 increased the protein level of HSD17B11.

**Conclusions:**

These data indicate that FTO may rely on the reading protein YTHDF1 to affect the translation pathway of the *HSD17B11* gene to regulate the formation of lipid droplets in EC cells, thereby promoting the development of EC. The understanding of the role of epitranscriptomics in the development of EC will lay a theoretical foundation for seeking new anticancer therapies.

**Supplementary Information:**

The online version contains supplementary material available at 10.1186/s13578-022-00798-3.

## Background

Recently, epitranscriptomics studies have contributed greatly to the development of research on human cancers. N6-methyladenosine (m6A) has become one of the most advanced and hot research topics in the life sciences field [1, 2]. Once the enzymes involved in m6A modification are abnormal, they will cause a series of diseases, including tumors [3–6], embryonic developmental delay [7], and neurological diseases [8]. Studies have shown that m6A methyltransferase plays a crucial role in the differentiation and self-renewal of mouse embryonic stem cells [9, 10]. Fat-mass and obesity-associated protein (FTO), which was the first discovered m6A demethylase, is involved in the regulation of adipocyte differentiation and cancer cell proliferation via the regulation of m6A modifications [11, 12]. It has been shown that changes in the expression of key genes sensitive to m6A regulatory enzymes can lead to substantial phenotypic changes. However, the biological significance and key target genes of these m6A regulatory enzymes in human cancer are still unclear.

Studies have shown that the number of lipid droplets (LDs) in breast cancer cells is related to their stemness and may be a potential target for cancer treatment [13]. LDs originate from the endoplasmic reticulum and are organelles that store lipids, mainly triglycerides and cholesterol esters. LDs contain enzymes that synthesize, store, utilize, and degrade a variety of lipids, playing an important role in lipid energy metabolism, and are the main regulators of lipid balance in cells [14]. Tirinato et al [15] demonstrated that high LD level was a special marker of cancer stem cells (CSCs) in colorectal cancer (CR), being directly related to the widely accepted colorectal cancer stem cell (CR-CSC) markers, such as CD133 and Wnt pathway. These results suggest that LD metabolites may promote the generation of tumor stem cell phenotypes. As a member of the short-chain dehydrogenase/reductase family, HSD17B11 family proteins have been reported to induce the aggregation of LDs, which regulate the dynamic changes of LDs and lipid metabolism by affecting LD-associated adipose triglyceride lipase [16]. However, the regulatory mechanism of LDs on esophageal cancer (EC) remains unclear.

In this study, we identified that FTO plays an important role in the progression of EC and promotes the proliferation, invasion, stemness, and tumorigenicity of EC cells (ECCs). Interestingly, sequence analysis revealed that HSD17B11 may be a target gene regulated by FTO. Moreover, FTO promoted the formation of LDs in ECCs by enhancing HSD17B11 expression. This study suggested that lipid metabolism is a novel molecular mechanism of cancer progression, which lays a theoretical foundation for the development of new anticancer therapies, so as to improve the prognosis of EC patients.

## Subjects and methods

### Patients and samples

The cancer and paracancerous tissue samples were collected in sterile conditions from 106 EC patients, placed in sterile enzyme-free tubes, fixed using paraformaldehyde, and embedded in paraffin for subsequent immunohistochemical detection. All tissue specimens were collected with the informed consent of the donors and the approval of the Ethics Committee of the First Affiliated Hospital of Zhengzhou University (2018-KY-92). The detailed information from patients is shown in Additional file 1: Table S1.

### Cell culture

The esophageal carcinoma cell lines KYSE510 and TE1 were purchased from Shanghai Institute of Cell Research, Chinese Academy of Sciences, and were cultured in endotoxin-free RPMI1640 medium (HyClone, USA) with 10% fetal bovine serum (Gibco, USA), penicillin (100 U/mL), and streptomycin (100 μg/mL) at 37 °C in a 5% CO2 incubator (Thermo Fisher Scientific, USA).

### Immunohistochemistry

Immunohistochemistry was used to determine the FTO expression level in EC tissue samples. The methods followed have been described in detail in a paper published by our research group [17]. Anti-FTO (1:500, Abcam) was used as the primary antibody. The slices were then imaged using a professional microscope (P-MIDI, 3D HISTECH, Hungary).

### RNA isolation and real-time quantitative PCR

Total RNA was extracted from the cell lines using TRIzol reagent. For cDNA synthesis, 1.0 μg of total RNA was used for reverse transcription in 10 μL of reaction volume using the PrimeScript RT reagent kit with gDNA Eraser (RR047A, Takara). Real-time quantitative PCR was performed using SYBR Green Premix Pro Taq HS qPCR kit (AG11701, Accurate Biotechnology, Human, Co., Ltd.) and a real-time PCR system (Applied Biosystem). *GAPDH* was used as the reference gene and each reaction was run in triplicates. In this study, we detected the mRNA expression levels of genes, namely *FTO*, *ALKBH5*, *CD44*, *CD90*, *CD133*, *CD271*, *OCT4*, *Bmi1*, *SOX2*, *SOX9*, *Nanog*, *KLF4*, *Nestin*, *YTHDF1*, and *HSD17B11*. All the primers used are listed in Additional file 1: Table S2.

### Determination of the total methylation level

We used the colorimetric method to measure the total m6A levels in mRNA using an EpiQuik m6A RNA Methylation Quantification kit (P-9005-96, Epigentek, USA). In this assay, the total RNA was bound to strip wells using an RNA high binding solution. m6A was detected using capture and detection antibodies. The detected signal was enhanced and then quantified colorimetrically by reading the absorbance on a microplate spectrophotometer. The amount of m6A is proportional to the OD intensity. Finally, we performed the relative quantification of the total m6A levels among different groups.

### Flow cytometry analysis

Flow cytometry was used to determine the expression levels of the main stem cell markers on the surface of ECCs, as previously described in detail [18]. The antibodies used were FITC anti-human CD90 (328108, BioLegend), PE anti-human CD133 (372804, BioLegend), and APC anti-human CD271 (345108, BioLegend).

### MeRIP and UID mRNA sequencing

The combined MeRIP and UID mRNA sequencing was performed between the FTO overexpression group and the vector group using esophageal carcinoma cell line (KYSE510), with the help of Seqhealth Technology Co., LTD (Wuhan, China). MeRIP and UID mRNA sequence data have been deposited in the Sequence Read Archive under the Bioproject ID PRJNA809833.

Briefly, the steps used to construct a library were the following: firstly, we used oligo-d (T) magnetic beads to enrich the mRNA, and used divalent cations to break the mRNA into short fragments of approximately 100bp. Next, m6A primary antibody was used for immune co-precipitation, and a strand of cDNA was synthesized using a random 6-base primer and using the RNA obtained from the immune co-precipitation as a template. We used a splint adapter to connect, and used magnetic beads to purify the product, and carried out PCR amplification. After the library was qualified, the different libraries were sequenced using an Illumina sequencer according to the effective concentration and target data volume requirements.

Raw sequencing data was first filtered using Trimmomatic (version 0.36). The data were mapped to *Homo-sapiens* genome version GRCh38 (ftp://ftp.ensembl.org/pub/release-87/fasta/homo_sapiens/dna/)**.** The exome Peak (version 3.8) software was used for peak calling. The bed tools (version 2.25.0) was used to annotate the m6A peaks. The peak distribution was analyzed using deep tools (version 2.4.1). Fisher exact test was used to identify the differential windows between the IP and input samples. The HOMER (version 4.10) software was used to identify the sequence motifs in m6A peak regions. Gene ontology (GO) analysis for annotated genes was implemented using the KOBAS software (version 2.1.1) with a corrected *P*-value cutoff of 0.05.

### RNA-mediated interference

We performed transient knockdown of *FTO* and *YTHDF1* expression in human esophageal carcinoma cell lines. The human-specific siRNA 01, siRNA 02, and siRNA 03 targeting the FTO gene and a negative control were purchased from GenePharma (Shanghai). The sequences of three siRNAs were as follows: siRNA-01, 5′-GUGGCAGUGUACAGUUAUA-3′; siRNA-02, 5′-CAGGAACCUUGGAUUAUAU-3′; siRNA-03, 5′-GCUGUGCUUCAUGAAGUUA-3′. The sequence of the human-specific siRNA targeting the *YTHDF1* gene was 5′-CCGCGTCTAGTTGTTCATGAA-3′. Esophageal carcinoma cell lines were transfected with siRNA (800 nM) using transfection reagent Lipofectamine 3000 (Thermo Fisher Scientific, USA).

### Plasmids and lentiviral transduction for stable cell lines

The human-specific shRNA lentiviral plasmids (carrier number: GV248, component order: hU6-MCS-Ubiquitin-EGFP-IRES-puromycin) targeting *FTO* or *YTHDF1* gene and a negative control were constructed by GK Gene Company (Shanghai) and stored in the form of a bacterial liquid. The sequences of the four shRNAs were as follows: FTO shRNA-1, 5′-CTAGAAGGAGCACAAGTCTCA-3′; FTO shRNA-2, 5′-GTCCCAAGAAATCGTGAGAAT-3′; YTHDF1 shRNA-1, 5′-TACCTGCTCTTCAGCGTCAAT-3′; YTHDF1 shRNA-2, 5′-AACCTCCATCTTCGACGACTT-3′. The FTO overexpression lentiviral plasmid (Ubi-MCS-3FLAG-SV40-EGFP-IRES-puromycin) carried the full-length CDS region sequence of the human FTO gene (GeneBank ID: NM_001080432), and the negative control (vector) was an empty plasmid without targeted DNA fragments. These were purchased from GK Gene Company (Shanghai). The methods used for plasmid extraction, lentivirus packaging, and virus transfection have been described in our previous study [19]. In this study, jetPRIME transfection reagent (PolyPlus, France) was used to transfect KYSE510 and TE1 cells with the plasmids.

### Cell counting kit-8 (CCK-8) and colony formation assays

CCK-8 (Dojindo, Japan) was used to detect the cell proliferation levels.

Tumor cells (200 per well) were cultured in a 37 °C, 5% CO2 incubator. After approximately 2 weeks of culture, the cell colonies were stained with 0.05% crystal violet for 20 min and counted using the microscope. The methods followed have been described in detail in a paper published by our research group [20].

### Transwell and wound-healing assays

Transwell (8.0 μm pore size, Corning, USA) and wound-healing assays were used to detect the migration of tumor cells, which has been described detailedly in a previous paper [19].

### Sphere formation assay and limiting dilution assay

The spheres were cultured in DMEM/F12 (Sigma) containing fibroblast growth factor-basic (20 ng/mL; PeproTech, USA), epidermal growth factor (20 ng/mL; PeproTech, USA), B27 supplement (Gibco), and heparin (4 μg/mL). After 7 days of culture in 6-well ultra-low adhesion plates (5000 cells/well), the sphere number per well was counted under a microscope, and the sphere formation rate was calculated.

A limiting dilution assay was performed as previously described [21]. Dissociated tumor cells were seeded in 96-well ultra-low adhesion plates at a density of 5, 10, 20, 50, 100 or 200 cells per well, with eight replicate wells per dose group. After culturing for 7 days, we counted the number of wells in which tumor sphere had been formed per dose group, and used extreme limiting dilution analysis to calculate the sphere formation rate (http://bioinf.wehi.edu.au/software/elda/).

### Western blotting

The extraction of total protein was carried out according to the standard experimental procedure, and the detailed detection methods were reported previously [19].

The primary antibodies used were as follows: mouse anti-FTO antibody (1:1000, Abcam, UK, #ab92821), rabbit anti-YTHDF1 antibody (1:1000, Proteintech, Wuhan, #17479-1-AP), rabbit anti-HSD17B11 antibody (1:1000, Proteintech, Wuhan, #16303-1-AP), and mouse anti-β-actin (1:2000, Cell Signaling Technology, USA, 3700S). Subsequently, they were incubated with rabbit or mouse IgG antibodies (1:5000, Zhongshan Bridge, Beijing, ZB-2301 or ZB-2305).

### RNA Immunoprecipitation (RIP)

RIP assay was performed on KYSE510 by SeqHealth (Wuhan, China). The cells were lysed using cell lysis buffer. The 10% lysed sample was divided into "input", 80% was used for the immunoprecipitation with FTO antibody (Proteintech, Wuhan, #27226-1-AP) and named "IP", and 10% was incubated with rabbit IgG antibody as a negative control. Next, the IP efficiency was detected by Western blot. The RNA of input and IP was recovered using TRIzol reagent, and RNA concentration was measured with the Qubit 3.0 (ThermoFisher), and reversed by the PrimeScript RT reagent kit with gDNA Eraser. RT-PCR for HSD17B11 was conducted using the listed primers in Additional file 1: Table S2.

### Detection of LDs

LDs in esophageal cancer cells (ECCs) were detected using cell special oil red O (ORO) staining solution (Beijing Solarbio), which could can stain the smallest LDs. Each group of cells was fixed with an ORO fixative solution for 20 min. Next, the samples were washed with 60% isopropanol for 5 min and posteriorly with newly prepared ORO staining solution for 15 min. The nuclei were counterstained using Mayer hematoxylin for 1 min. After incubation with ORO buffer for 1 min, the cells were covered with distilled water and observed using a microscope.

### Xenograft nude mouse model

To generate a xenograft mouse model, healthy Balb/c nude mice were purchased from Beijing Weitong Lihua Laboratory Animal Technology Co., Ltd, aged 4–6 weeks, and weighing 18–20 g. The breeding conditions met the SPF standards and were approved by the Animal Ethics Committee of Zhengzhou University. Nude mice were randomly divided into five groups of six mice each, and the mice in each group were received subcutaneous injections of shFTO, scrambled shNC, FTO OE, and vector KYSE510 cells (5×10^6^ tumor cells/mouse), respectively. The tumor size and weight of mice were measured 1 week later, which was recorded as day 0, and then measured once every other day; and the tumor volumes were calculated using the following formula: (length×width^2^)/2. When the tumor maximum diameter was close to 15 mm, the mice were euthanized and the tumor tissues were collected for immunohistochemistry analysis. We conducted the xenograft mouse model experiments in the Henan Key Laboratory for Pharmacology of Liver Diseases, and all the animal experiments were approved by the Ethics Committee of the First Affiliated Hospital of Zhengzhou University.

### Statistical analysis

SPSS21.0 and GraphPad Prism 7 software were used for the statistical analysis of experimental data. For the quantitative data, Student t test and analysis of variance (ANOVA) were used for those that conformed to a normal distribution, and the rank sum test was used for the data that were inconsistent with a normal distribution. For the qualitative data, chi-square test was used to compare the differences between two groups. The Kaplan–Meier method was used for survival analysis. Univariate and multivariate Cox regression analyses were used to analyze the factors affecting the survival of EC patients. Two-sided testing, *α*=0.05 is the test level.

## Results

### FTO is elevated in EC stem-like cells and is a poor prognostic factor for EC patients

**Fig. 1:**
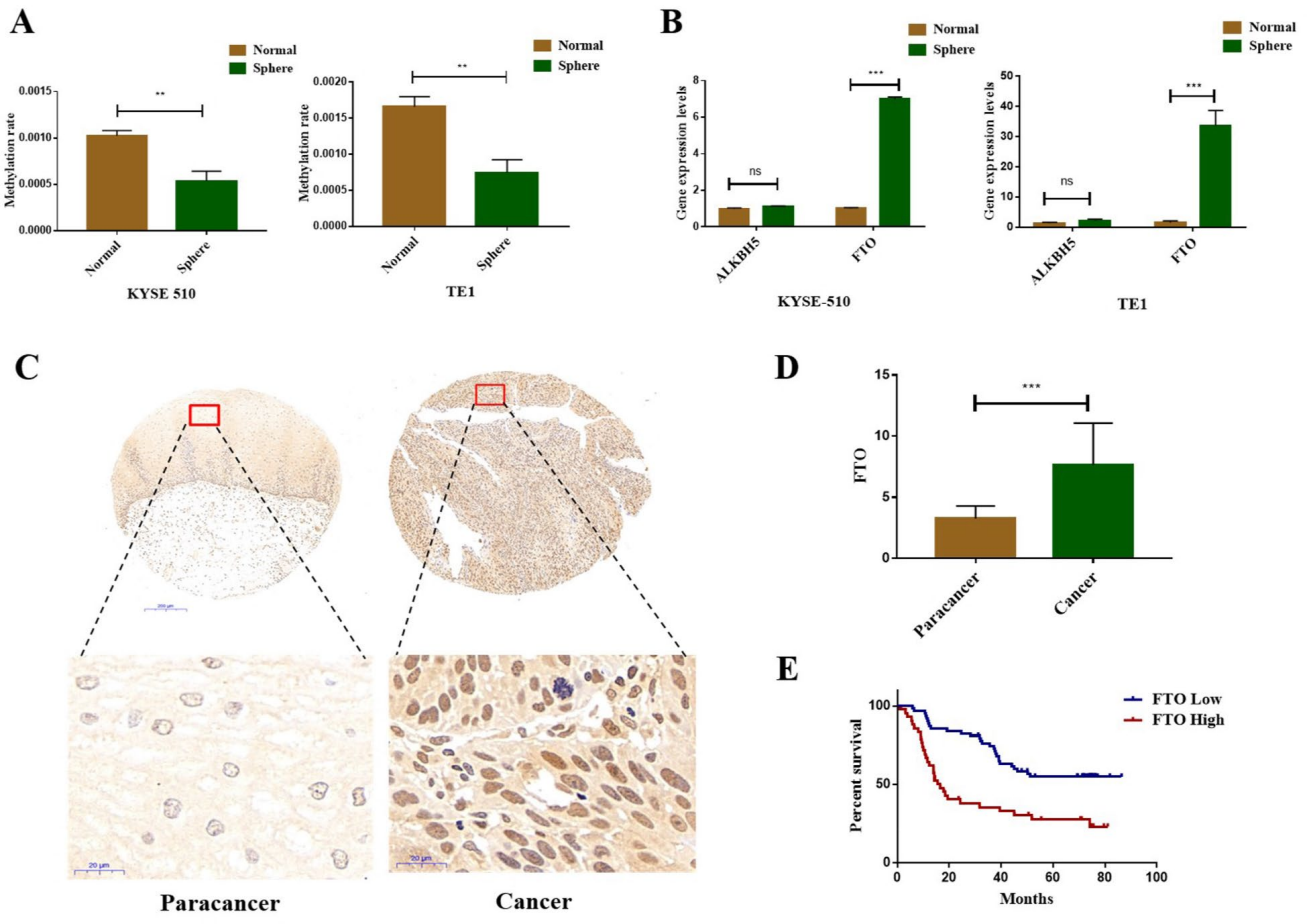
FTO is elevated in esophageal cancer stem-like cells and is a poor prognostic factor in EC patients. **A** EpiQuik m6A RNA Methylation Quantification kit (Colorimetry) was used to detect the total methylation levels of mRNA extracted from normal or sphere cells; **B** qPCR analysis of ALKBH5 and FTO mRNA expression levels between normal and sphere cells; **C** Immunohistochemical analysis of FTO protein in paracancerous and cancer tissues of EC patients; **D** Statistical results of the FTO protein levels in paracancerous and cancer tissues of EC patients (t=11.27, *P* < 0.001); **E** Kaplan-Meier survival curve was used to analyze the overall patient survival in groups with a high and low FTO expression. Log-rank test was used to compare the median overall survival duration between the two groups (*x*^*2*^=15.233, *P* < 0.001).

It has been shown that the existence of CSCs is a key factor that leads to tumor relapse and proliferation [22]. Therefore, we explored the specific gene expression for this subset of tumor cells. We used ultra-low adhesion plates to culture the EC cell lines into spheres to enrich the tumor stem-like cells (Additional file 2: Fig. S1A). Furthermore, we found that there was a significantly increasing trend in the expression levels of stemness-related markers and cell renewal factors in the tumor spheres (*P* < 0.05) (Additional file 2: Fig. S1B–S1D), indicating that the enriched stem-like cells were successfully induced. Many studies have shown that RNA methylation plays an important role in regulating the tumor stem-like properties [23–25]. Thus, we detected the total methylation level of RNA in tumor stem-like cells and found a significantly decreasing trend (*P* < 0.05) (Fig. 1A). Therefore, we detected the mRNA expression levels of demethylation genes *ALKBH5* and *FTO* between tumor stem-like cells and their matching cell lines. *FTO* expression was evidently increased in tumor stem-like cells (*P* < 0.05); however, there was no significant change in the expression level of ALKBH5 (*P* > 0.05) (Fig. 1B). These results indicate that FTO has been associated with the stemness of ECCs.

Furthermore, we collected 106 cancer tissues and their paired paracancerous tissues from EC patients. The general characteristics of the subjects are shown in Additional file 1: Table S1. The protein level of FTO in the above-mentioned tissues was detected via immunohistochemistry (Fig. 1C), which showed that FTO expression in EC tissues was significantly increased compared to that in paracancerous tissues (Fig. 1D). Collectively, FTO is a critical determinant of tumor progression.

Thereafter, we analyzed the relationships between gender, age, pathological grade, tumor stage, lymph node metastasis, tumor size, and the FTO expression level (Additional file 1: Table S3). We divided the EC patients into two groups based on the median of FTO expression level; the statistical results showed that only lymph node metastasis was related to the expression level of FTO (*P* < 0.05), whereas the other variables had no statistical differences (*P* > 0.05).

Previously, we found that the expression level of demethylase FTO was significantly higher in EC stem-like cells, and many studies have shown that the tumor stem cells could promote the development and metastasis of cancers [26]. Next, we analyzed the prognostic value of FTO and the clinical parameters in EC patients using Kaplan–Meier (KM) survival curve analysis. The results showed that the overall survival of patients was significantly reduced in the FTO high expression group (Fig. 1E). Patients in the high-age (> 60-years old) group had a poor prognosis. However, the gender and pathological grade had no effect on the overall survival of patients. Further analysis found that the patients with the higher tumor stage and lymph node metastasis had a worse prognosis (Additional file 2: Fig. S1E). Furthermore, we used univariate and multivariate COX regression to perform further statistical analysis (Additional file 1: Table S4). The results of univariate COX analysis showed that the age, tumor stage, lymph node metastasis, and FTO expression level had an effect on the overall survival of EC patients. Higher age, higher tumor stage, lymph node metastasis, and higher FTO expression were adverse factors for patient survival, which were consistent with the results of the KM curve analysis. We further analyzed the poor prognostic factors using multivariate COX regression. After adjusting for some factors, such as pathological grade, gender, and so on, we found that age, tumor stage, and FTO expression levels still affected the overall survival of EC patients. All these findings demonstrate that FTO is related to the occurrence and development of EC.

### Blockade of FTO decreases the proliferation, migration, and stemness of ECCs

To analyze the effect of FTO on ECCs, transient FTO-knockdown TE1 cells (siFTO cells) were constructed; FTO knockdown efficacy in TE1 cells was analyzed using qPCR (Additional file 3: Fig. S2A). After FTO transient knockdown, the expression levels of the representative stem cell genes CD90, CD133, and SOX2 were significantly decreased (Additional file 3: Fig. S2B), and the cell migration ability in TE1 was also significantly weakened, as observed in the wound-healing and Transwell assays (Additional file 3: Fig. S2C, S2D).

**Fig. 2:**
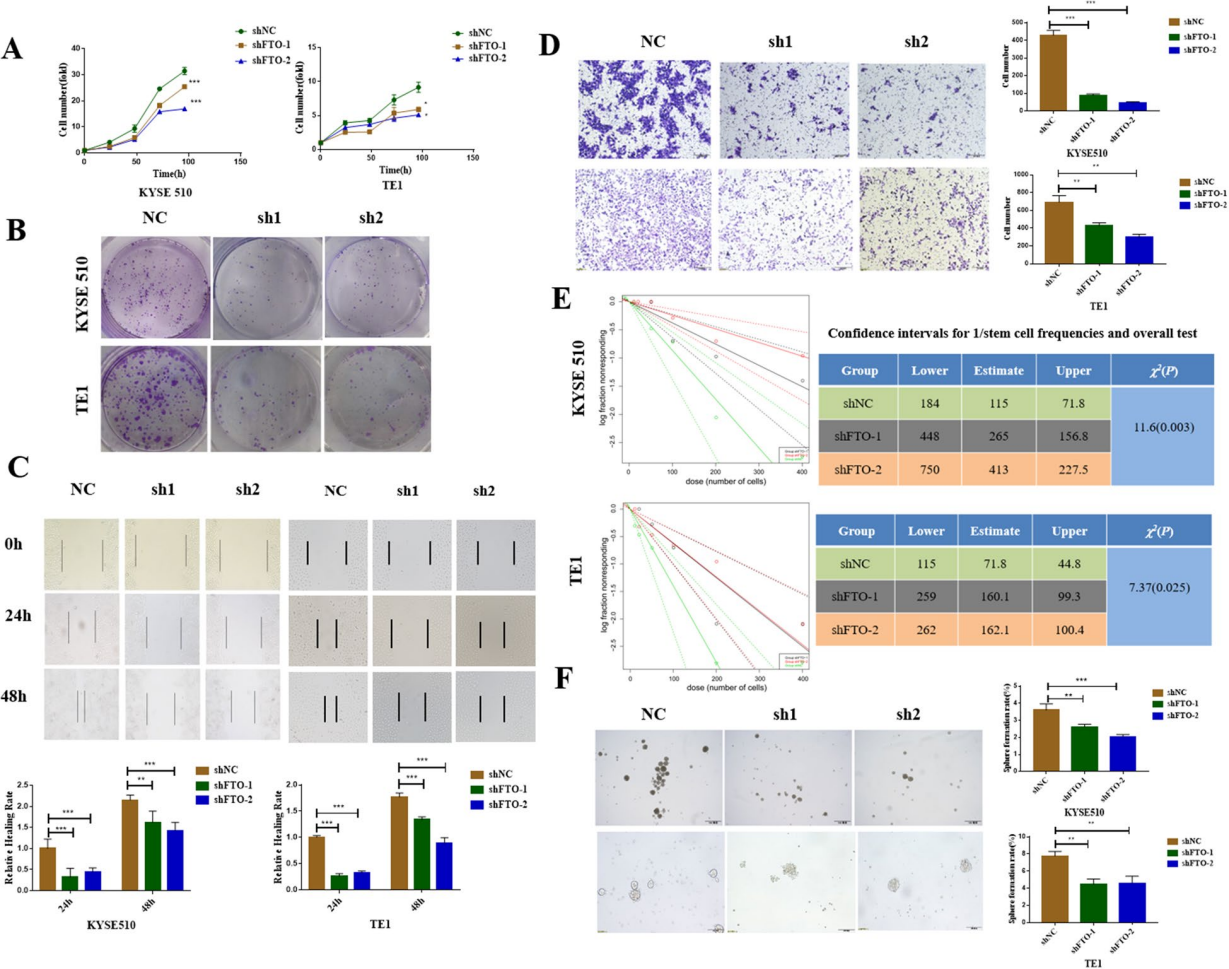
FTO inhibition decreases the proliferation, migration, and stemness of ECCs. **A** CCK-8 assay was used to detect the cell proliferation in ECCs with or without FTO knockdown; **B** Cell colony formation was used to detect the long-term proliferation of ECCs with or without FTO knockdown; **C** Cell migration results of wound-healing assays, and the **D** Transwell assay performed using ECCs with or without FTO knockdown and the respective statistical results; **E** Using extreme limiting dilution assay analysis, the frequency of stem cells in each group was estimated with 95% confidence intervals, and the differences among the groups were analyzed using chi-square test; **F** Sphere formation ability of shFTO and shNC ECCs, the histograms on the right represent the statistical results. ^*^P < 0.05, ^**^P < 0.01, ^***^P < 0.001.

Next, we used two short hairpin RNAs (shFTO-1 and shFTO-2) to ablate FTO expression. We obtained the stable FTO-knockdown cell lines through lentiviral transfection, flow sorting, and puromycin screening (Additional file 4: Fig. S3A). The results showed that two short hairpin RNAs significantly inhibited the mRNA and protein expression levels of FTO (Additional file 4: Fig. S3C, S3E). FTO knockdown could decrease the cell proliferation and colony formation ability of ECCs (Fig. 2A, B), and decrease the cell migration ability, as observed in the wound-healing and Transwell assays (Fig. 2C, D). Moreover, FTO knockdown significantly decreased the spheroidization ability, as observed in the vitro limiting dilution and sphere formation assays (Fig. 2E, F). Collectively, FTO plays an important role in tumor progression and might be a potential therapeutic target in EC.

### FTO overexpression promotes the proliferation, migration, and stemness of ECCs

**Fig. 3:**
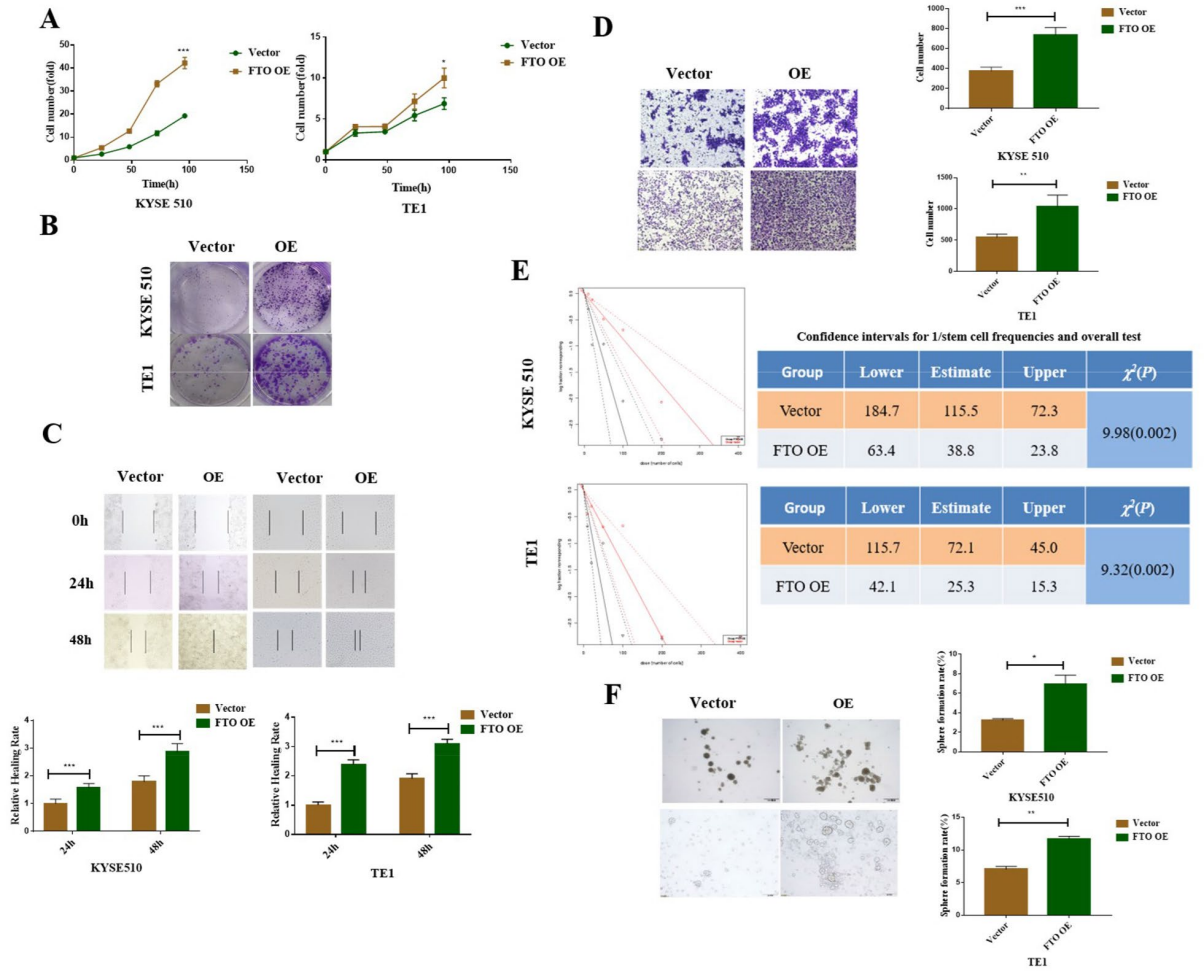
FTO overexpression promotes the proliferation, migration, and stemness of ECCs. **A** CCK-8 assay was used to detect cell proliferation of ECCs in the negative control and FTO overexpression groups; **B** Cell colony formation assays were used to detect the long-term proliferation of ECCs in the negative control and FTO overexpression groups; **C** Cell migration results of wound-healing assays, and the **D** Transwell assay in the negative control and FTO overexpression groups and the respective statistical results; **E** Using extreme limiting dilution assay analysis, the FTO overexpression group was shown to have an increased spheroidization ability compared to the control group; the frequency of stem cells in each group was estimated using 95% confidence intervals, and the difference between the two groups was analyzed using chi-square test; **F** Sphere formation ability of FTO OE and vector ECCs, the histograms on the right represent the statistical results. ^*^P < 0.05,^**^P < 0.01, ^***^P < 0.001.

To further verify the effect of FTO expression on the biological behavior of ECCs, we constructed ECCs overexpressing FTO through lentiviral transfection, flow sorting and puromycin screening (Additional file 4: Fig. S3B), and studied the effect of FTO overexpression on the proliferation, migration, and spheroidization ability of ECCs. FTO overexpression efficacy was determined using qPCR and western blotting (Additional file 4: Fig. S3D, S3F). The results suggested that FTO overexpression could significantly increase the cell proliferation, colony formation, cell migration, and spheroidization abilities of ECCs (Fig. 3A–F), which were completely opposite to the results obtained from FTO knockdown.

### FTO enhances the tumorigenicity of ECCs *in vivo*

**Fig. 4:**
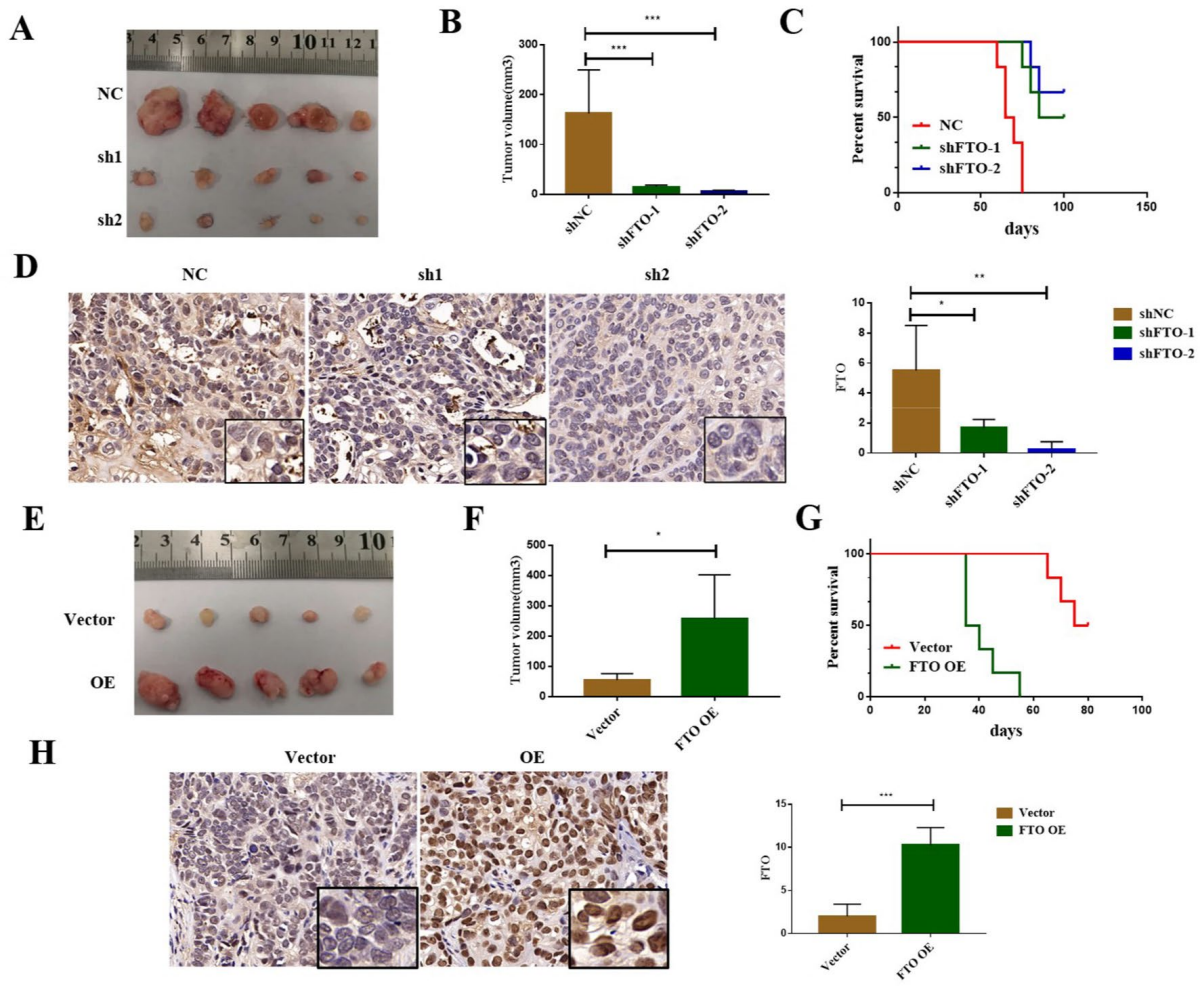
The FTO expression level influences the tumorigenesis of ECCs *in vivo*. **A** Representative images of subcutaneous tumors in immunodeficient Balb/c nude mice with shNC and shFTO ECCs; **B** Statistical results of the tumor volumes among the three groups; **C** Survival analysis of mice subcutaneously injected with the ECCs with or without FTO knockdown (*x*^*2*^*=*18.528,*P* < 0.001);**D** Immunohistochemical detection of the FTO expression levels in subcutaneous tumors with or without FTO knockdown and statistical results; **E** Representative images of the subcutaneous tumors in immunodeficient Balb/c nude mice with FTO OE and vector ECCs; **F** Statistical results of the tumor volumes between the negative control and FTO overexpression groups; **G** Survival analysis of the mice subcutaneously injected with the esophageal cancer cell lines with FTO OE and vector (*x*^*2*^*=*12.429, *P* < 0.001); **H** Immunohistochemical detection of the FTO expression levels in the subcutaneous tumors derived from the injection of the negative control and FTO overexpression into mice and statistical results; *P < 0.05, **P < 0.01, ***P < 0.001.

Next, we validated the effect of FTO depletion on the tumorigenicity via subcutaneous injection of shNC or shFTO ECCs into nude mice. Compared with the shNC group, the tumor volume was significantly reduced in nude mice injected with shFTO cells (Fig. 4A, B), and FTO knockdown remarkably prolonged the survival of the corresponding tumor-bearing mice (*P*<0.001) (Fig. 4C), and the FTO protein level was significantly decreased in the tumor tissues compared with the control group (Fig. 4D). Furthermore, we validated the effect of FTO on the tumorigenicity using ECCs overexpressing FTO. Contrary to the results obtained from the stable FTO knockdown, the overexpression of FTO gene promoted the growth of transplanted tumors in mice and shortened the survival of the tumor-bearing mice (Fig. 4E–G). Moreover, the FTO protein level was significantly increased in the tumor tissues of FTO overexpression group (Fig. 4H). Therefore, the demethylation activity of FTO is critical for the tumorigenicity of ECCs.

### Analysis of the downstream targets of FTO in ECCs

**Fig. 5:**
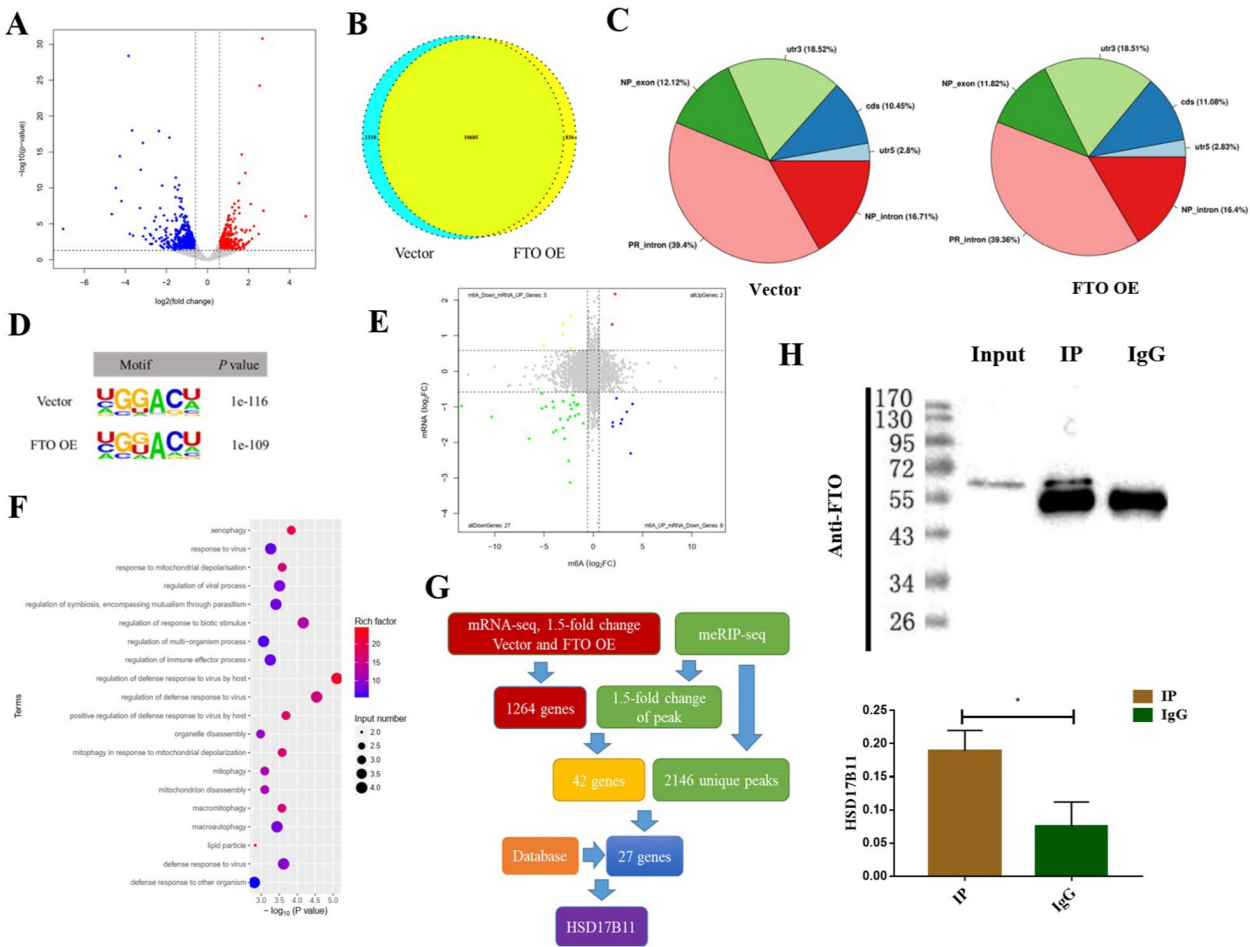
Analysis of the downstream targets of FTO in ECCs. **A** Volcano map of the gene expression from UID mRNA-seq data between the negative control and FTO overexpression groups; The abscissa: log2 (fold change); the ordinate: − log10 (p-value); Gray dots represent the genes that were not differentially expressed; the blue dots represented the genes that were differentially down-regulated, and the red dots represented the genes that were differentially up-regulated; **B** Venn diagram of the peak associated genes of meRIP-seq data between the negative control and FTO overexpression groups; **C** The distribution of the peaks in each functional area of the gene; **D** Top consensus motif identified by HOMER with meRIP-seq peaks in ECCs with or without FTO overexpression; **E** Four-quadrant diagram of the meRIP-seq differential genes and mRNA-seq differential genes; **F** GO Analysis for the genes with reduced methylation and mRNA levels. **G** Schematic diagram of the screening of downstream target genes; **H** RNA Immunoprecipitation assay was used to detect the binding between HSD17B11 and FTO protein in KYSE510 cells (The upper band is the targeted band, and the lower band is the antibody heavy chain), and statistical results.

To explore the molecular mechanism of demethylase FTO in regulating the occurrence and development of EC, we performed combined sequencing of meRIP-seq and UID mRNA-seq in the KYSE510 cells overexpressing FTO and the vector group. For the UID mRNA-seq data, a total of 1264 genes were differentially expressed by at least 1.5-fold change between the two groups, and *P-*value was less than 0.05, including 423 up-regulated and 841 down-regulated genes (Fig. 5A). Regarding the meRIP-seq data, the Venn diagram of the peaks showed that there were a total of 10,685 peaks in common, 1,310 unique peaks in the vector group and 836 unique peaks in the FTO overexpression group (Fig. 5B). Moreover, the m6A modification peaks were mainly concentrated in the intron region of the genes (Fig. 5C). The m6A consensus motif GGACU was identified in ECCs with or without FTO overexpression (Fig. 5D). Next, we conducted a joint analysis of meRIP-seq and mRNA-seq and used the four-quadrant diagram to display a total of 42 differential genes; among them, there were 27 genes that all decreased in m6A and mRNA levels, 5 genes with a decrease of m6A level and an increase of mRNA level, 2 genes that all increased in m6A and mRNA levels, and 8 genes with an increase of m6A level and a decrease of mRNA level (Fig. 5E). As FTO is a demethylase, we searched for the downstream targets of FTO among the genes with a decreased methylation level, that were enriched in the regulation pathway of LDs using GO analysis (Fig. 5F). Through geneplot analysis, the methylation modified site of targeted gene HSD17B11 in this study was found in the coding region of the gene (Additional file 5: Fig. S4A). We finally determined that *HSD17B11* could be the downstream target of FTO, based on a literature search and database analysis, and that the reading protein YTHDF1 might be involved in this regulation (Additional file 5: Fig. S4B). The specific process used for the analysis is shown in Fig. 5G. Next, we determined the mRNA expression level of HSD17B11 in cells with an FTO overexpression, and it exhibited a decreasing trend (Additional file 5: Fig. S4C), which is consistent with the sequencing results. After transient YTHDF1 knockdown in KYSE510 cells, the mRNA expression level of HSD17B11 was also decreased (Additional file 5: Fig. S4D), which is consistent with the result predicted by the database. Furthermore, RNA Immunoprecipitation assay was to validate the binding between HSD17B11 and FTO protein in KYSE510 cells, and the result showed that FTO proteins could bind to mRNA of targeted gene HSD17B11 (Fig. 5H). All these data indicate that HSD17B11 gene is associated with FTO gene in ECCs.

### FTO regulates lipid metabolism by relying on the reading protein YTHDF1

**Fig. 6:**
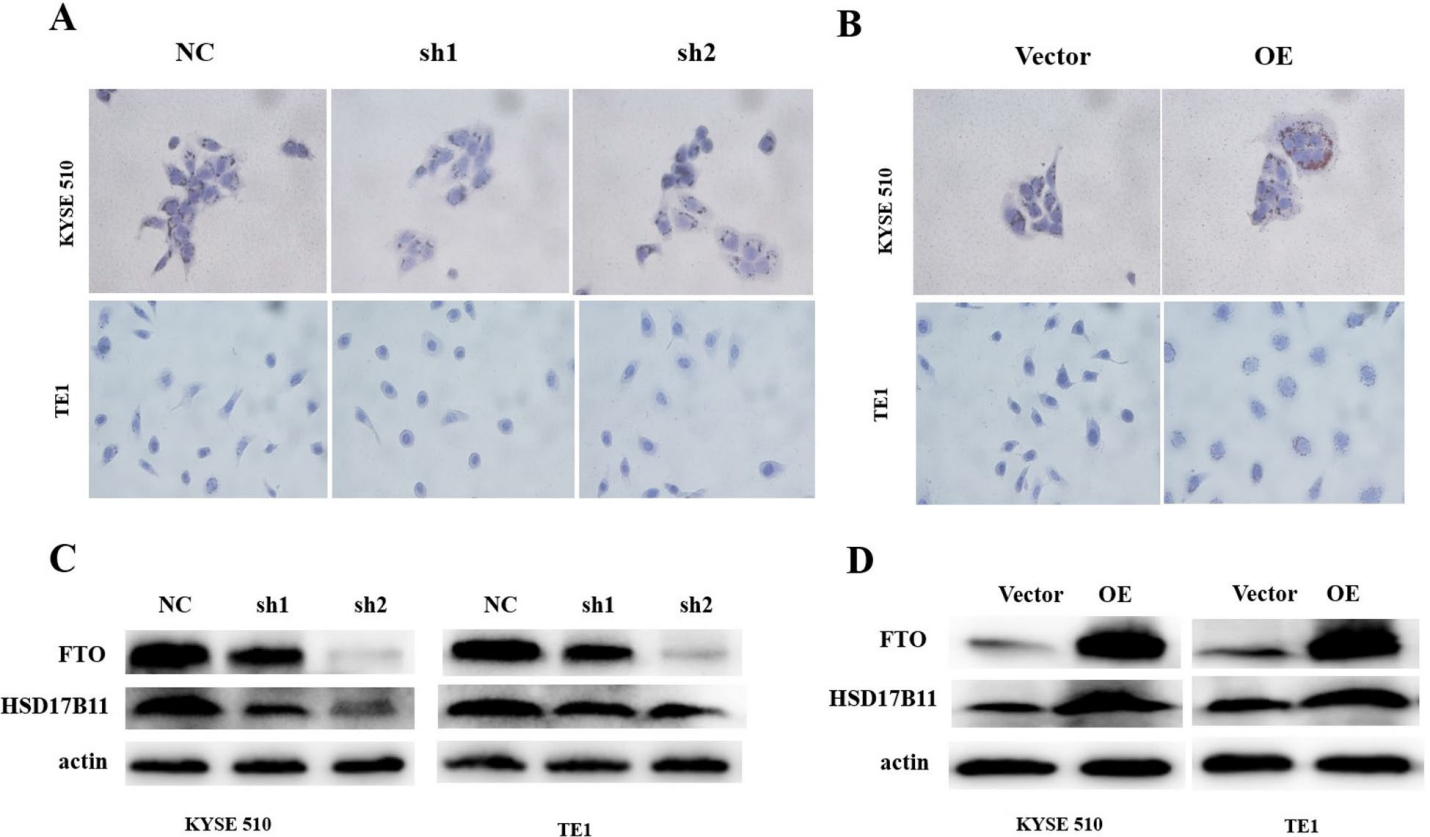
FTO influences the tumor lipid metabolism by regulating the HSD17B11 expression level. **A** Oil red O staining method was used to detect the lipid droplets in the ECCs with or without FTO knockdown; **B** Representative pictures of the lipid droplets in the ECCs with or without FTO overexpression; **C** Western blotting of HSD17B11 in the ECCs with or without FTO knockdown, actin served as an internal control; **D** Western blotting of HSD17B11 in ECCs with or without FTO overexpression.

The above results showed that FTO could regulate lipid metabolism by targeting HSD17B11. Next, we used oil red O staining to detect the effects of FTO on LDs in ECCs. The results showed that the number of LDs was significantly decreased after stable FTO knockdown (Fig. 6A), and obviously increased after FTO overexpression (Fig. 6B). Furthermore, we detected the HSD17B11 protein expression level in the lipid droplet regulation pathway using western blotting, which showed a significantly decreasing or increasing trend with the knockdown or overexpression of FTO (Fig. 6C, D).

**Fig. 7:**
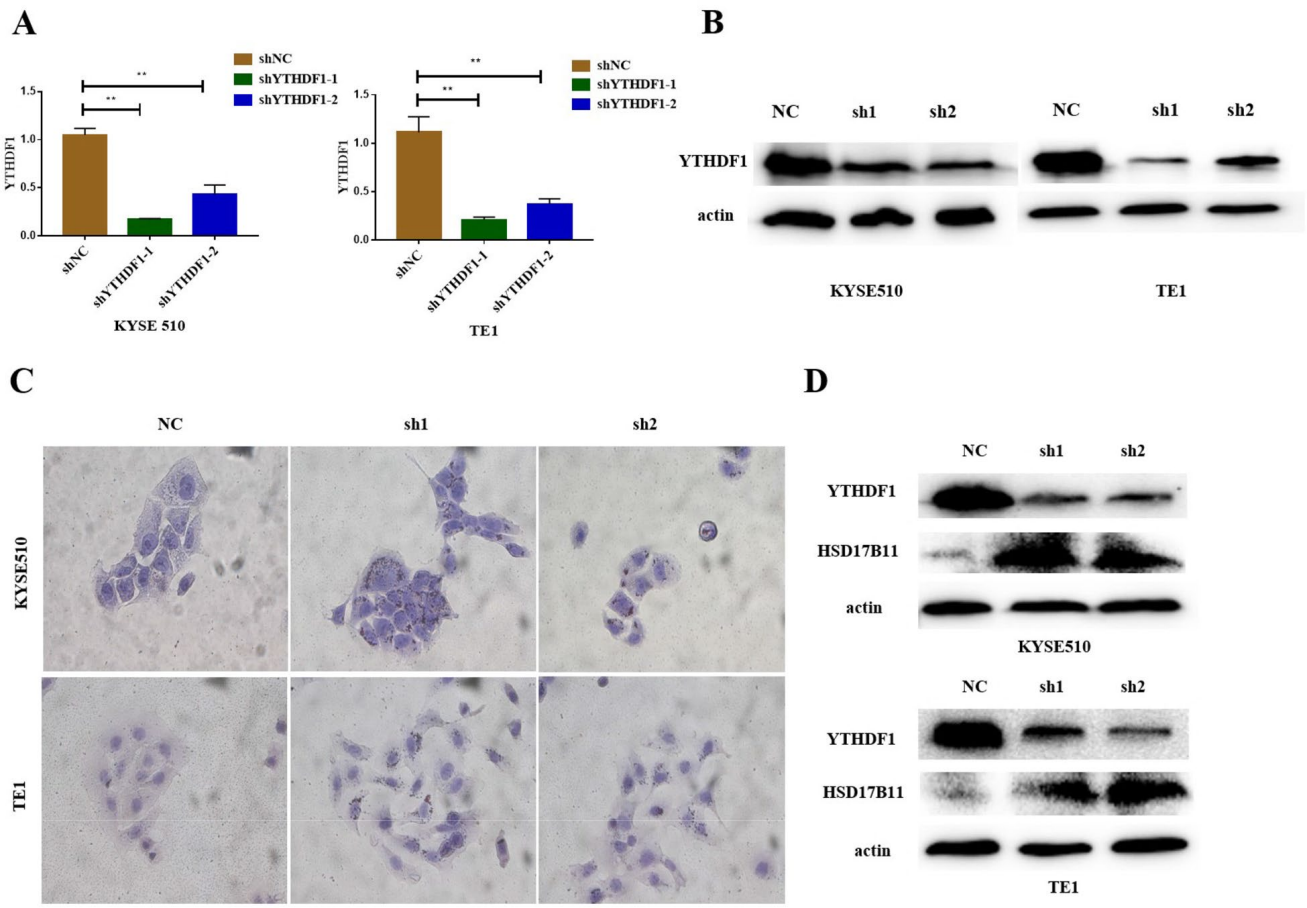
FTO regulates lipid metabolism by relying on YTHDF1 protein. **A** qPCR analysis of the knockdown efficiency of *YTHDF1* gene in ECCs; **B** Western blotting of YTHDF1 knockdown efficiency; **C** Oil red O staining method was used to detect the lipid droplets in the ECCs with or without YTHDF1 knockdown; **D** Western blotting of HSD17B11 in the ECCs with or without YTHDF1 knockdown.

Previous studies have shown that YTHDF1 can affect the translation ability of mRNA [27]. Thus, we used two short hairpin RNAs (shYTHDF1-1 and shYTHDF1-2) to ablate YTHDF1 expression in ECCs. We obtained the stable YTHDF1-knockdown cell lines through lentiviral transfection, flow sorting, and puromycin screening, and the results showed that the two shRNAs significantly inhibited the mRNA and protein expression of YTHDF1 (Fig. 7A, B). YTHDF1 knockdown could increase the number of LDs in ECCs (Fig. 7C). Furthermore, the HSD17B11 protein expression level in ECCs also showed a significant increase with the YTHDF1 knockdown (Fig. 7D). The results of this study are consistent with previous reports [16], that high expression level of HSD17B11 could promote the aggregation of LDs.

## Discussion

FTO was originally reported to be associated with weight gain and obesity in humans, and studies have shown that it regulates lipid metabolism in an m6A-dependent manner [28, 29]. It is widely expressed in all adult and fetal tissues and is highly expressed in the brain [30]. As an m6A demethylase, FTO eliminates m6A through a two-step reaction, first converting m6A into N6-hydroxymethyladenosine, then converting it into N6-formyladenosine, and finally into A [31]. FTO has been shown to be carcinogenic in both leukemia and glioblastoma [12, 32]. However, a study on ovarian cancer showed that the FTO gene inhibited ovarian cancer stem cell self-renewal and tumor initiation [33]. In the current study, we found that the total methylation level was significantly decreased, and the demethylation gene FTO showed a significantly high expression in EC stem-like cells. It was further confirmed that the stable knockdown of FTO significantly inhibited the proliferation, migration, stemness, and tumorigenicity of ECCs, whereas FTO overexpression promoted the above characteristics, which suggests that FTO may be a new target in tumor therapy.

In HepG2 cells, *FTO* overexpression increased the expression levels of lipid metabolism genes (*FASN*, *SCD1*, and *MGAT1*), but decreased the expression levels of lipid transport genes (*MTTP*, *APOB*, and *LIPC*), resulting in lipid accumulation; the *FTO* R316A mutant lacking demethylase activity did not result in these effects [29]. Next, we performed combined sequencing of meRIP-seq and UID mRNA-seq and analyzed the regulation pathway enriched to LDs using GO analysis. Through the database analysis and validated assays, we finally determined that HSD17B11 could be a target gene that is regulated by FTO, and that reading protein YTHDF1 might be involved in this regulation. The results of this study showed that the HSD17B11 protein level showed an increasing trend in the lipid droplet regulation pathway after the stable knockdown of YTHDF1. Next, we detected the changes in the number of LDs in ECCs after the stable knockdown of YTHDF1, and the results showed that it was significantly increased, which is consistent with previous reports [16]. And previous studies have shown that YTHDF1 can affect the translation ability of mRNA [27]. This study shows that the reader protein YTHDF1 may regulate tumor lipid metabolism by reducing the translation efficiency of the target gene HSD17B11.

LDs are highly organized spherical organelles which are storage reservoirs of cellular fat. LDs develop in the endoplasmic reticulum via budding and are then transported to the cytoplasm [14, 34, 35]. The core of LDs is composed of neutral fats, mainly triglycerides and cholesterol esters, and is covered by a single layer of phospholipid molecules and various proteins. The phospholipid molecules are mainly phosphatidylcholine and phosphatidylethanolamine, followed by phosphatidylinositol [36]. In mammalian cells, there are proteins specifically located in LDs; mainly adipose differentiation-related protein, acyl-CoA synthetase 3, and 17beta-hydroxysteroid dehydrogenase 11 (HSD17B11) [37]. As a member of the short-chain dehydrogenase/reductase family, HSD17B11 family proteins has been reported to induce the aggregation of LDs, that regulates the dynamic changes of LDs and the lipid metabolism by affecting LD-associated adipose triglyceride lipase [16]. LDs have two different functions: one is to provide alternative energy when glycolysis is blocked, and the other is to protect fatty acids from the harmful effects of peroxidation [38]. In this study, we found that the protein expression level of HSD17B11 was significantly decreased or increased after the inhibition or overexpression of FTO, respectively. Moreover, the number of LDs in ECCs showed a decreasing or increasing trend with the inhibition or overexpression of FTO, respectively.

Many studies have shown that the number of LDs in CSCs is higher than that in differentiated tumor cells [15, 39]. In colorectal cancer, cells with a high level of LDs showed CSC tumorigenicity characteristics both *in vivo* and *in vitro*. High levels of LDs were positively correlated with the expression levels of CD133 and Wnt/β-catenin [15]. In addition, it has been reported that CSCs accumulate LDs as a lipid reserve for energy supply [40]. Singh et al. [41] found that blocking lipolysis by targeting the vesicle-mediated COPI complex, which transports lipase to the surface of LDs, resulted in the death of CSCs. These data indicate that LDs are very important for the maintenance of CSCs, but further studies are still needed to clarify their role and possible applications for the targeted therapy of CSCs.

In conclusion, this firstly reveals that HSD17B11 is the downstream target of FTO. Moreover, FTO promoted the formation of lipid droplets in EC cells by enhancing HSD17B11 expression. Furthermore, depleting YTHDF1 increased the protein level of HSD17B11. These findings indicate that FTO may rely on the reading protein YTHDF1 to affect the translation pathway of the HSD17B11 gene to regulate the formation of lipid droplets in EC cells, thereby promoting the development of EC.

## Conclusions

In this study, we found that FTO was a poor predictor in EC patients and elevated in EC stem-like cells. Further research showed that FTO promoted the occurrence and development of EC by mediating lipid metabolism, and HSD17B11 could be a key component in mediating FTO-dependent tumor stemness. These findings not only provide a novel insight into the molecular mechanisms underlying EC pathogenesis, but also open up avenues for seeking new anticancer therapies.

## Supplementary Information

**Additional file 1:  Table S1.** General characteristics of 107 patients with esophageal squamous cell carcinoma. **Table S2.** Primer sequences for detection genes. **Table S3.** The relationship between general characteristics and FTO expression level in ESCC patients. **Table S4.** Univariate and multivariate analyses of overall survival in ESCC patients

**Additional file 2: Figure S1.** Esophageal cancer stem-like cells and survival analysis. **A** Representative images of the cell spheres in the ECCs; **B** qPCR analysis of the cell stemness markers and cell renewal factors after sphering of ECCs; **C** Flow cytometry detected the cell stemness markers CD90, CD133, and CD271, **D** the corresponding statistical results are presented; **E** Kaplan-Meier survival curves were used to analyze the effects of age, sex, pathological grade, tumor stage, and lymph node metastasis on the overall patient survival.

**Additional file 3: Figure S2.** Transient knockdown of the FTO gene inhibits the migration of ECCs. **A** qPCR analysis of the knockdown efficiency of the FTO gene in siFTO cells; **B** qPCR analysis of representative cell stemness markers (CD90, CD133, CD271, and SOX2) with or without FTO transient knockdown; **C** Cell migration was analyzed using wound-healing assays with or without FTO transient knockdown and statistical results; **D** Cell migration was investigated using Transwell assays with or without FTO transient knockdown and statistical results (P < 0.001).

**Additional file 4: Figure S3.** Construction of ECCs with the stable knockdown and overexpression of FTO gene. **A**, **B** Flow cytometry was used to detect the transfection efficiency of the constructed stable knockdown (**A**) and overexpression (**B**) in ECCs; **C**, **D** qPCR analysis of the knockdown (**C**) and overexpression (**D**) efficiency of the FTO gene; **E**, **F** Western blotting of FTO in ECCs after FTO knockdown (**E**) and overexpression (**F**).

**Additional file 5: Figure S4.** The relationships among the genes. **A** Geneplot analysis of target gene HSD17B11, the methylation modified site of targeted gene HSD17B11 in this study was found in the coding region of the gene (marked in the red box); **B** Database analysis of the correlation between FTO, YTHDF1 and the target gene HSD17B11 (http://gepia.cancer-pku.cn/detail.php); **C** qPCR analysis of HSD17B11 mRNA expression between the negative control and FTO overexpression groups; **D** Changes in the mRNA expression level of HSD17B11 gene after YTHDF1 was knocked down transiently.

## Data Availability

The data and material in this study are available within the article.

## Abbreviations

m6A: N6-methyladenosine
EC: Esophageal cancer
FTO: Fat mass and obesity-associated protein
LDs: Lipid droplets
CSCs: cancer stem cells
ECCs: Esophageal cancer cells
ANOVA: Analysis of variance
KM: Kaplan-Meier
shRNAs: Short hairpin RNAs
HSD17B11: 17Beta-hydroxysteroid dehydrogenase 11
