# Supplementary material for: m6A demethylase FTO promotes tumor progression via regulation of lipid metabolism in esophageal cancer
Source: Cell Biosci. 2022 May 14;12:60. doi: 10.1186/s13578-022-00798-3 (PMC9107638; doi:10.1186/s13578-022-00798-3)
Supplement: Supplementary file 1 — Additional file 1: Table S1. General characteristics of 107 patients with esophageal squamous cell carcinoma. Table S2. Primer sequences for detection genes. Table S3. The relationship between general characteristics and FTO expression level in ESCC patients. Table S4. Univariate and multivariate analyses of overall survival in ESCC patients [file 13578_2022_798_MOESM1_ESM.docx]

Additional file 1: Table S1. General characteristics of 107 patients with esophageal squamous cell carcinoma

| Variables | Patient Numbers (%) |
| --- | --- |
| Age(years) |  |
| ＜60 | 53(50%) |
| ≥60 | 53(50%) |
| Gender |  |
| Male | 87(82.1%) |
| Female | 19(17.9%) |
| Grade |  |
| Higher | 22(20.8%) |
| Middle | 58(54.7%) |
| Lower | 26(24.5%) |
| Tumor stage |  |
| Ⅰ | 23(21.7%) |
| Ⅱ | 36(34.0%) |
| Ⅲ | 41(38.7%) |
| Ⅳ | 6(5.7%) |
| Lymph node metastasis |  |
| Yes | 50(47.2%) |
| No | 56(52.8%) |

Additional file 1: Table S2. Primer sequences for detection genes

| Gene | | Primer Sequence | length（bp） |
| --- | --- | --- | --- |
| FTO | F | 5’-GCTGCTTATTTCGGGACCTG-3’ | 84 bp |
|  | R | 5’-AGCCTGGATTACCAATGAGGA-3’ |  |
| ALKBH5 | F | 5’-ATGCACCCCGGTTGGAAAC-3’ | 250 bp |
|  | R | 5’-GACTTGCGCCAGTAGTTCTCA-3’ |  |
| CD44 | F | 5’-CTGCCGCTTTGCAGGTGTA-3’ | 109 bp |
|  | R | 5’-CATTGTGGGCAAGGTGCTATT-3’ |  |
| CD90 | F | 5’-ATCGCTCTCCTGCTAACAGTC-3’ | 135 bp |
|  | R | 5’-CTCGTACTGGATGGGTGAACT-3’ |  |
| CD133 | F | 5’-AGTCGGAAACTGGCAGATAGC-3’ | 99 bp |
|  | R | 5’-GGTAGTGTTGTACTGGGCCAAT-3’ |  |
| CD271 | F | 5’-CCGTTGGATTACACGGTCCAC-3’ | 239 bp |
|  | R | 5’-TGAAGGCTATGTAGGCCACAA-3’ |  |
| OCT4 | F | 5’-CCCCTGGTGCCGTGAAG-3’ | 97 bp |
|  | R | 5’-GCAAATTGCTCGAGTTCTTTCTG-3’ |  |
| Bmi1 | F | 5’-CCACCTGATGTGTGTGCTTTG-3’ | 162 bp |
|  | R | 5’-TTCAGTAGTGGTCTGGTCTTGT-3’ |  |
| SOX2 | F | 5’-TGGACAGTTACGCGCACAT-3’ | 215 bp |
|  | R | 5’-CGAGTAGGACATGCTGTAGGT-3’ |  |
| SOX9 | F | 5’-AGCGAACGCACATCAAGAC-3’ | 85 bp |
|  | R | 5’-CTGTAGGCGATCTGTTGGGG-3’ |  |
| Nanog | F | 5’-CAAAGGCAAACAACCCACTT-3’ | 158 bp |
|  | R | 5’-TCTGCTGGAGGCTGAGGTAT-3’ |  |
| KLF4 | F | 5’-CCCACATGAAGCGACTTCCC-3’ | 170 bp |
|  | R | 5’-CAGGTCCAGGAGATCGTTGAA-3’ |  |
| Nestin | F | 5’-CAACAGCGACGGAGGTCTC-3’ | 164 bp |
|  | R | 5’-GCCTCTACGCTCTCTTCTTTGA-3’ |  |
| YTHDF1 | F | 5’-ACCTGTCCAGCTATTACCCG-3’ | 99 bp |
|  | R | 5’-TGGTGAGGTATGGAATCGGAG-3’ |  |
| HSD17B11 | F | 5’-TGCACATTTCTGGACTACAAAGG-3’ | 176 bp |
|  | R | 5’-CAGCCAGTTCATCTGTCAAAGT-3’ |  |
| GAPDH | F | 5’-GCACCGTCAAGGCTGAGAAC-3’ | 138 bp |
|  | R | 5’-TGGTGAAGACGCCAGTGGA-3’ |  |

Note: F: upstream primer, R: downstream primer.

Additional file 1: Table S3. The relationship between general characteristics and FTO expression level in ESCC patients

| Variables | High  （n=43） | Low  （n=63） | *x^2^***/***t***/**Z | *P* |
| --- | --- | --- | --- | --- |
| Age Grouping |  |  |  |  |
| ＜60 | 22 (51.2) | 31 (49.2) | 0.039^b^ | 0.843 |
| ≥60 | 21 (48.8) | 32 (50.8) |  |  |
| Age(years) | 60.12±10.946 | 59.68±9.858 | 0.213^a^ | 0.832 |
| Gender |  |  |  |  |
| Male | 37(86.0) | 50(79.4) | 0.775 | 0.379 |
| Female | 6(14.0) | 13(20.6) |  |  |
| Grade |  |  |  |  |
| Higher | 10(23.3) | 12(19.0) | 4.378 | 0.112 |
| Middle | 27(62.8) | 31(49.2) |  |  |
| Lower | 6(14.0) | 20(31.7) |  |  |
| Tumor stage |  |  |  |  |
| Ⅰ+Ⅱ | 21(48.8) | 38(60.3) | 1.365 | 0.243 |
| Ⅲ+Ⅳ | 22(51.2) | 25(39.7) |  |  |
| Lymph node metastasis |  |  |  |  |
| Yes | 26(60.5) | 24(38.1) | 5.132 | 0.023^†^ |
| No | 17(39.5) | 39(61.9) |  |  |
| Tumor sizes | 3.71±1.46 | 3.92±1.40 | 0.732^a^ | 0.466 |

^a^ *t* test.

^b^$\chi$^2^ test.

^†^Statistical differences.

Additional file 1: Table S4**.** Univariate and multivariate analyses of overall survival in ESCC patients

| Variables | Univariate Analysis | | |  | Multivariate Analysis | | |
| --- | --- | --- | --- | --- | --- | --- | --- |
|  | *β* | HR (95% CI) | *P* |  | *β* | HR (95% CI) | *P* |
| Age(years) | 0.033 | 1.034(1.008-1.061) | 0.011^†^ |  | 0.029 | 1.029(1.005-1.055) | 0.019^†^ |
| Gender | -0.094 | 0.910(0.472-1.753) | 0.778 |  |  |  |  |
| Grade | 0.246 | 1.279(0.859-1.904) | 0.226 |  |  |  |  |
| Tumor stage | 0.513 | 1.671(1.220-2.288) | 0.001^†^ |  | 0.485 | 1.624(1.088-2.424) | 0.018^†^ |
| Lymph node metastasis | 0.675 | 1.963(1.170-3.293) | 0.011^†^ |  |  |  |  |
| Tumor sizes | -0.016 | 0.984(0.829-1.169) | 0.856 |  |  |  |  |
| FTO expression | 0.193 | 1.213(1.117-1.316) | <0.001^†^ |  | 0.191 | 1.210(1.111-1.318) | <0.001^†^ |

^†^Statistical differences.
