# Supplementary figures and images for: m6A demethylase FTO promotes tumor progression via regulation of lipid metabolism in esophageal cancer
Source: Cell Biosci. 2022 May 14;12:60. doi: 10.1186/s13578-022-00798-3 (PMC9107638; doi:10.1186/s13578-022-00798-3)

**A**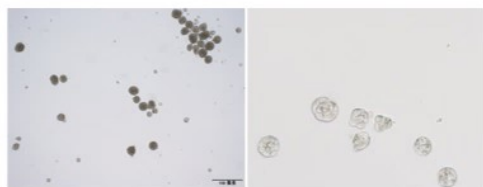

KYSE 510

TE1

**B**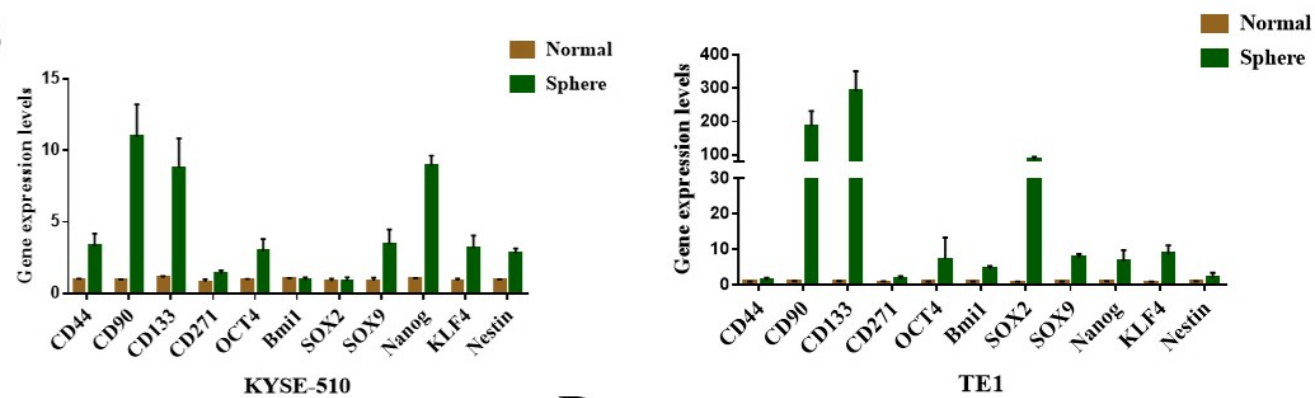

KYSE-510

TE1

**C**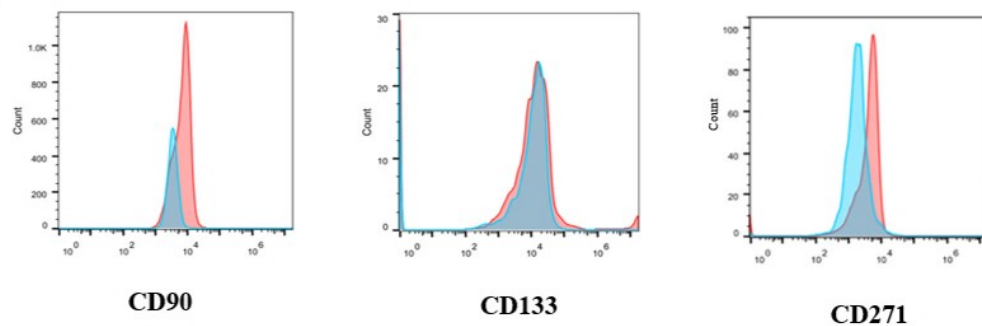

CD90

CD133

CD271

**D**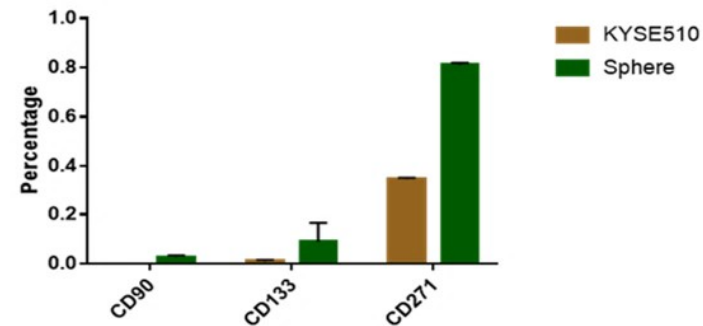**E**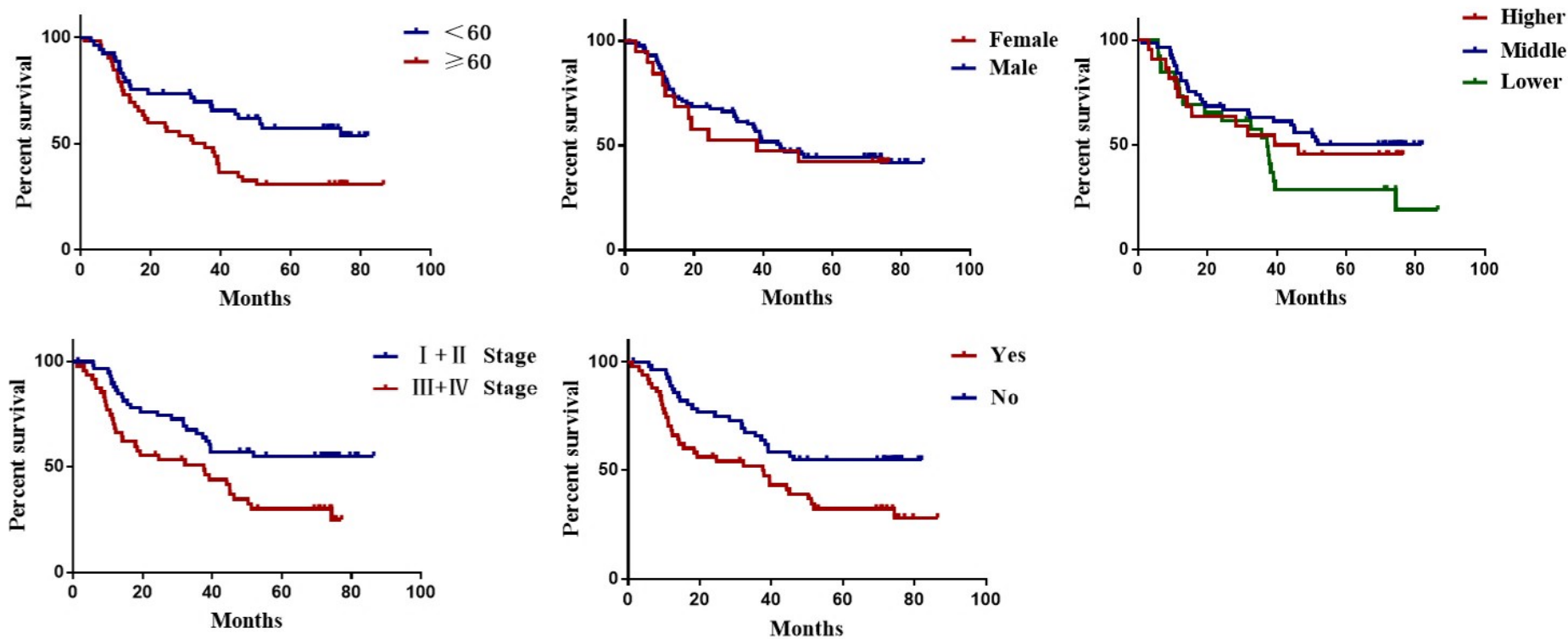

Supplement: Supplementary file 2 — Additional file 2: Figure S1. Esophageal cancer stem-like cells and survival analysis. A Representative images of the cell spheres in the ECCs; B qPCR analysis of the cell stemness markers and cell renewal factors after sphering of ECCs; C Flow cytometry detected the cell stemness markers CD90, CD133, and CD271, D the corresponding statistical results are presented; E Kaplan-Meier survival curves were used to analyze the effects of age, sex, pathological grade, tumor stage, and lymph node metastasis on the overall patient survival. [file 13578_2022_798_MOESM2_ESM.pdf]

**A**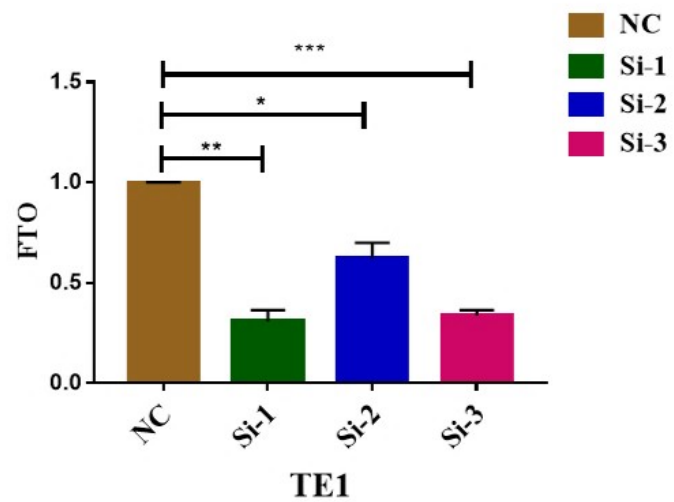**B**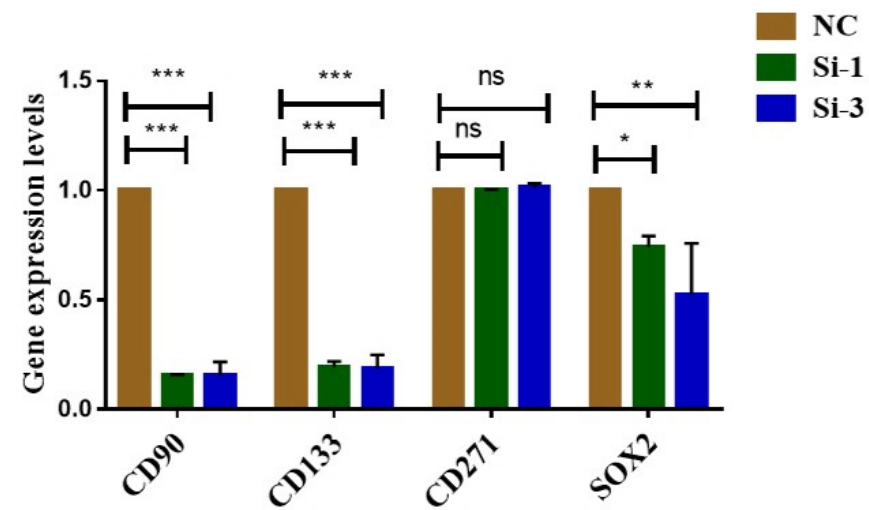**C**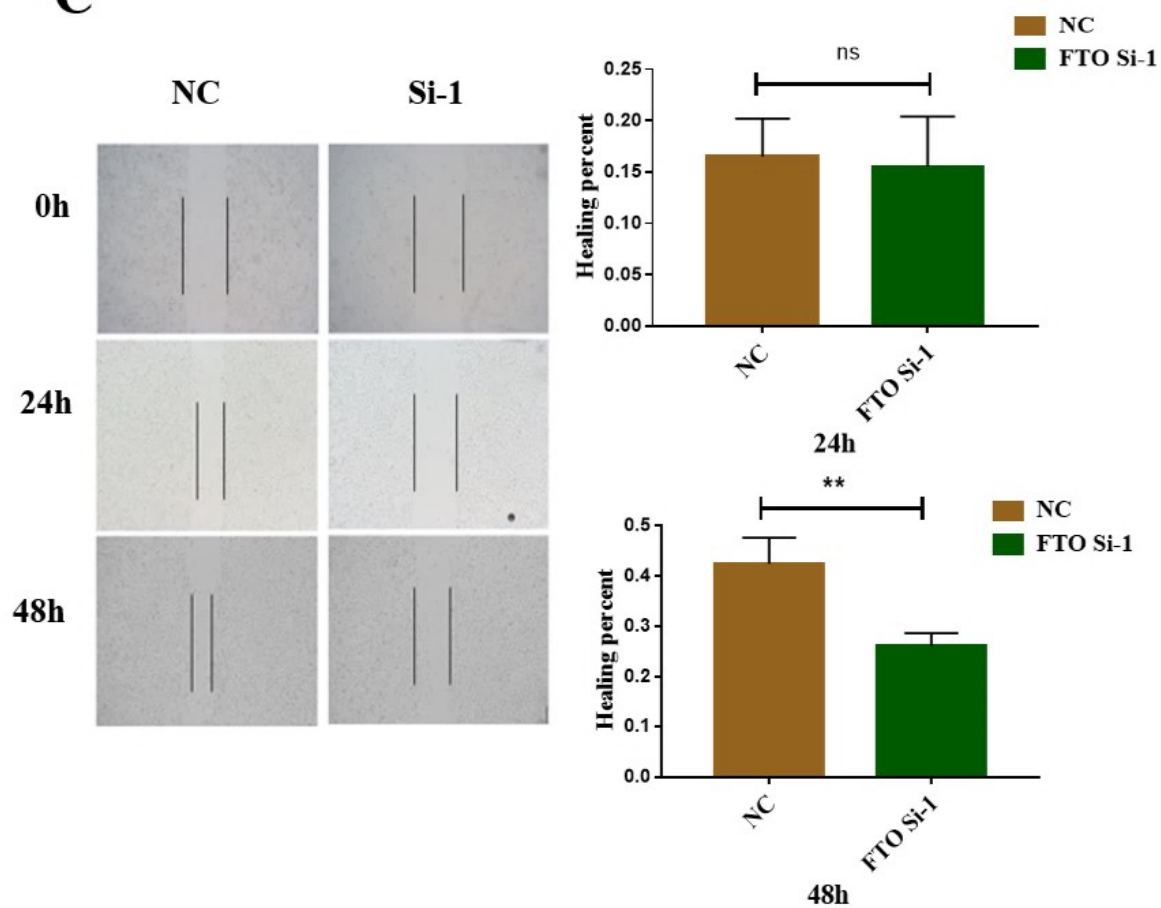**D**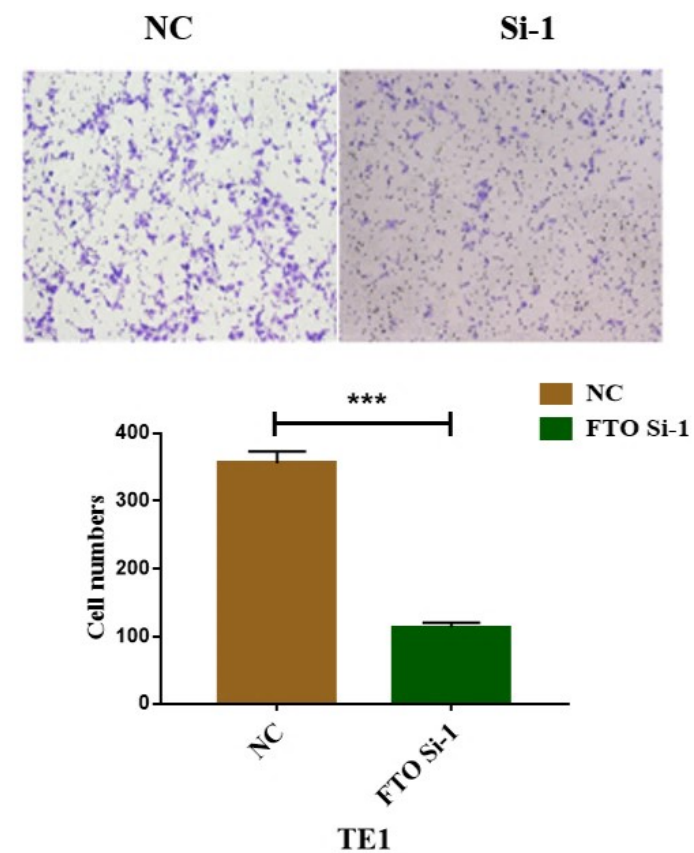

Supplement: Supplementary file 3 — Additional file 3: Figure S2. Transient knockdown of the FTO gene inhibits the migration of ECCs. A qPCR analysis of the knockdown efficiency of the FTO gene in siFTO cells; B qPCR analysis of representative cell stemness markers (CD90, CD133, CD271, and SOX2) with or without FTO transient knockdown; C Cell migration was analyzed using wound-healing assays with or without FTO transient knockdown and statistical results; D Cell migration was investigated using Transwell assays with or without FTO transient knockdown and statistical results (P < 0.001). [file 13578_2022_798_MOESM3_ESM.pdf]

**A**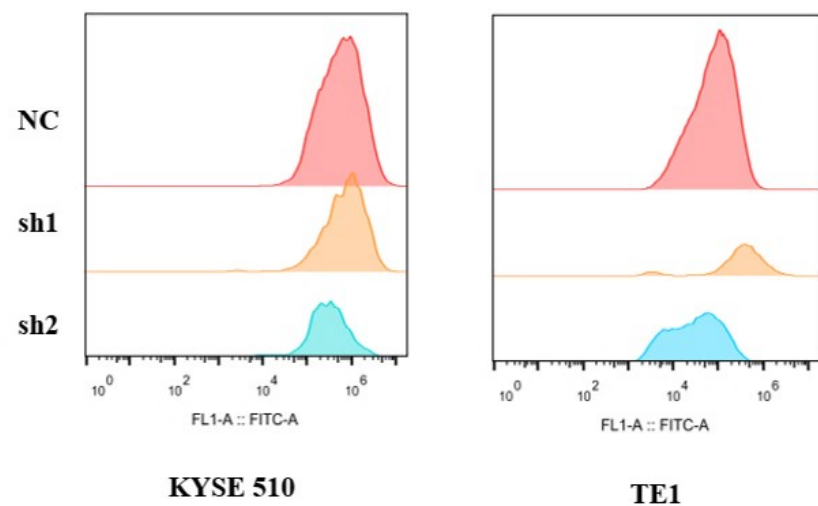**B**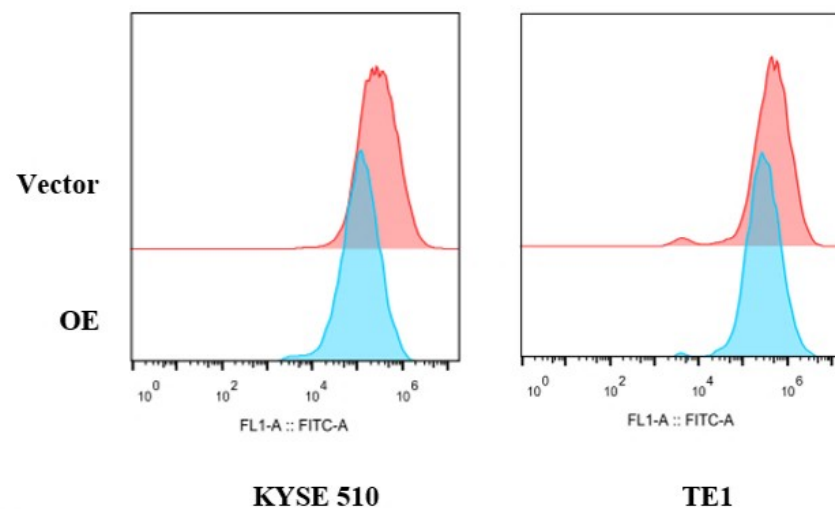**C**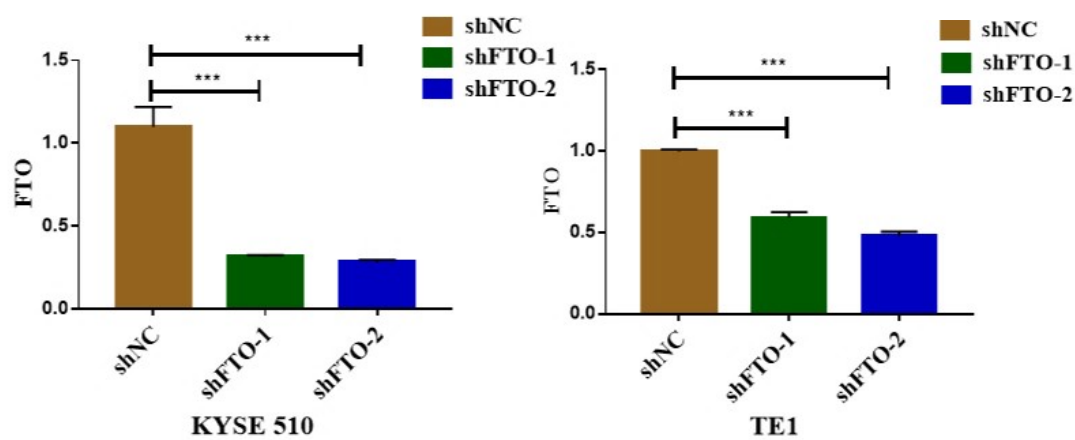**D**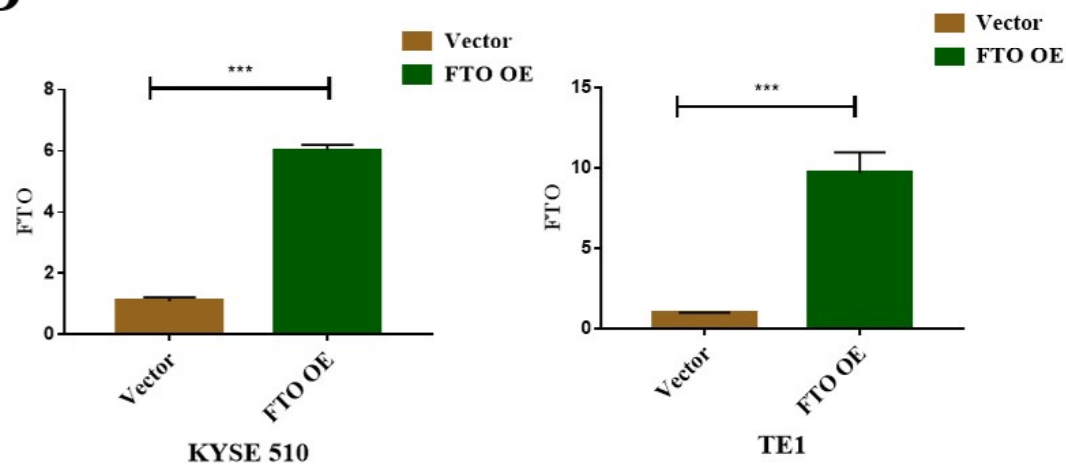**E**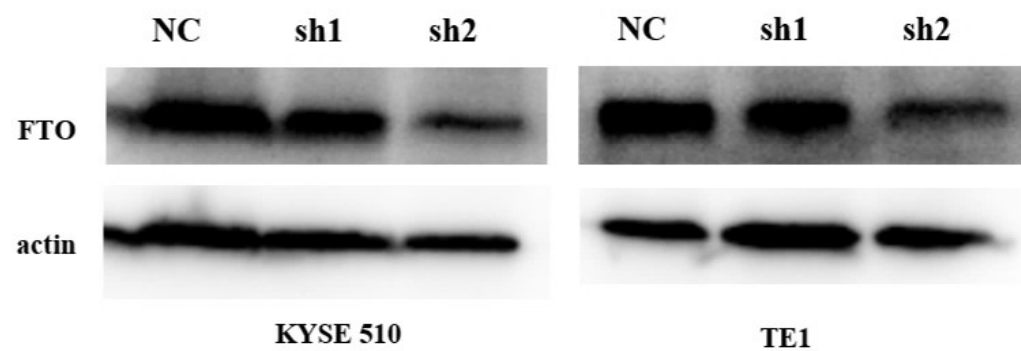**F**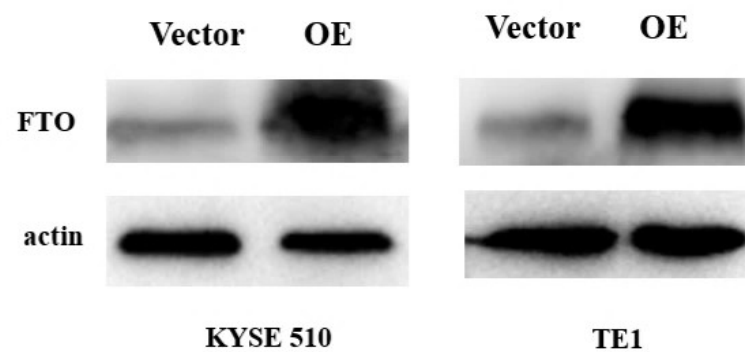

Supplement: Supplementary file 4 — Additional file 4: Figure S3. Construction of ECCs with the stable knockdown and overexpression of FTO gene. A, B Flow cytometry was used to detect the transfection efficiency of the constructed stable knockdown (A) and overexpression (B) in ECCs; C, D qPCR analysis of the knockdown (C) and overexpression (D) efficiency of the FTO gene; E, F Western blotting of FTO in ECCs after FTO knockdown (E) and overexpression (F). [file 13578_2022_798_MOESM4_ESM.pdf]

**A**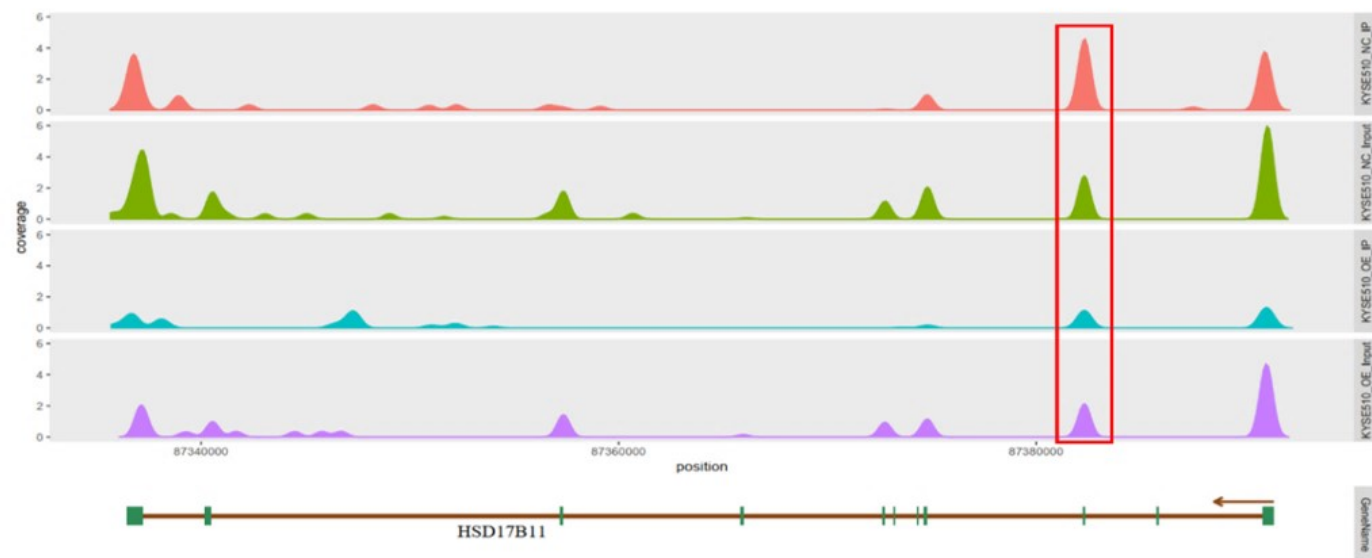**C**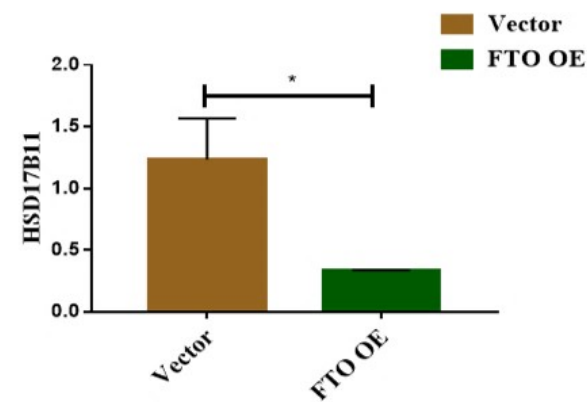**B**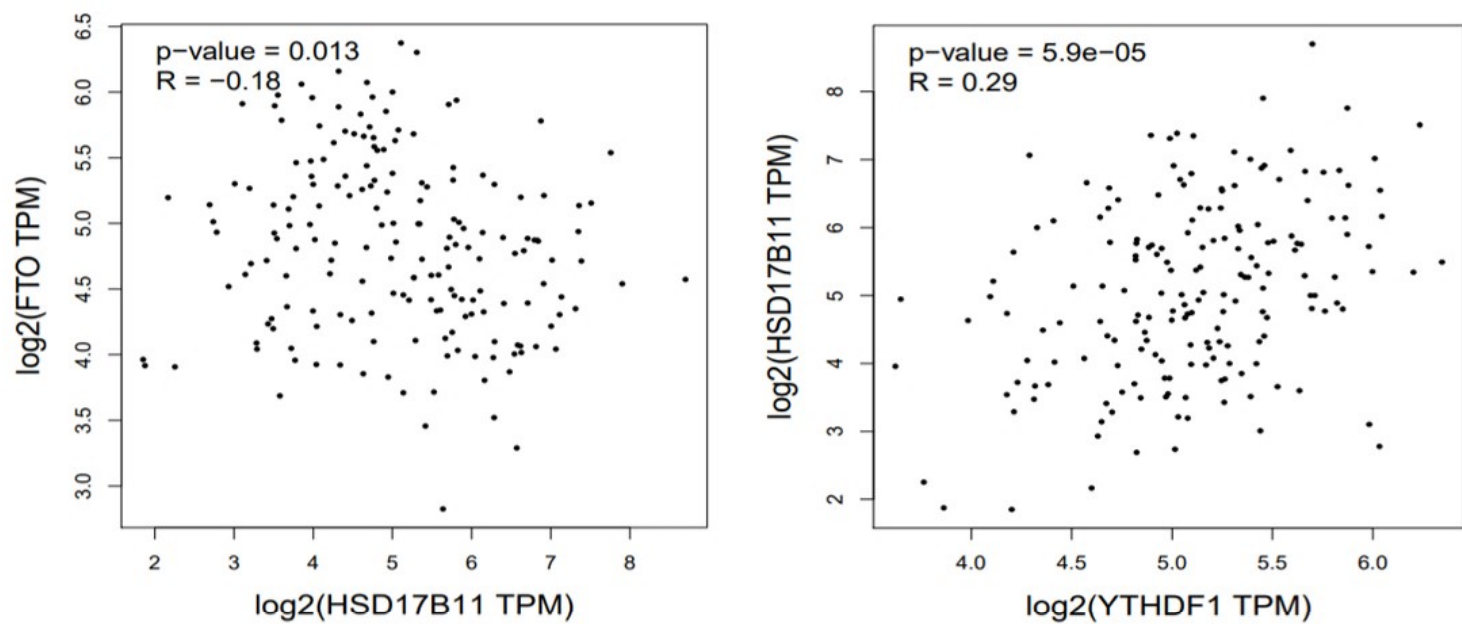**D**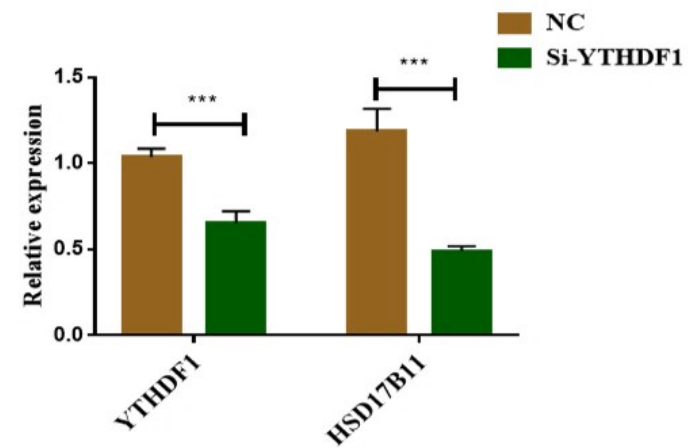

Supplement: Supplementary file 5 — Additional file 5: Figure S4. The relationships among the genes. A Geneplot analysis of target gene HSD17B11, the methylation modified site of targeted gene HSD17B11 in this study was found in the coding region of the gene (marked in the red box); B Database analysis of the correlation between FTO, YTHDF1 and the target gene HSD17B11 (http://gepia.cancer-pku.cn/detail.php); C qPCR analysis of HSD17B11 mRNA expression between the negative control and FTO overexpression groups; D Changes in the mRNA expression level of HSD17B11 gene after YTHDF1 was knocked down transiently. [file 13578_2022_798_MOESM5_ESM.pdf]
